# A new species of *Nicippe* from the Bering Sea (Crustacea, Amphipoda, Pardaliscidae), with a redescription of *N. tumida*

**DOI:** 10.3897/zookeys.965.51749

**Published:** 2020-09-03

**Authors:** Ko Tomikawa, Keiichi Kakui, Yoshihiro Fujiwara

**Affiliations:** 1 Department of Science Education, Graduate School of Education, Hiroshima University, Higashi-Hiroshima 739-8524, Japan Hiroshima University Higashi-Hiroshima Japan; 2 Faculty of Science, Hokkaido University, Sapporo, Hokkaido 060-0810, Japan Hokkaido University Sapporo Japan; 3 Marine Biodiversity and Environmental Assessment Research Center, Japan Agency for Marine-Earth Science and Technology, Yokosuka, Kanagawa 237-0061, Japan Japan Agency for Marine-Earth Science and Technology Yokosuka Japan

**Keywords:** COI, deep sea, *Nicippe beringensis*, systematics

## Abstract

A new pardaliscid amphipod, *Nicippe beringensis*, is described from the Bering Sea at depths between 520 and 536 m, and *N. tumida* Bruzelius, 1859 is redescribed based on specimens from Fredrikshald, Norway, near the species’ type locality. Nucleotide sequences of mitochondrial cytochrome *c* oxidase subunit I (COI) from the holotype and a paratype of *N. beringensis***sp. nov.** are reported. While *N. beringensis***sp. nov.** is similar to *N. tumida*, it differs from the latter in having an asetose palp article 1 of maxilla 1, a gnathopod 1 coxa with a straight distal edge, and in the posterior margin of the basis of gnathopods 1 and 2, and pereopod 3, being heavily setose in females. A key to species of *Nicippe* is provided.

## Introduction

The genus *Nicippe* Bruzelius, 1859 has been recorded from littoral to deep-sea marine environments; it currently comprises five species: *N. tumida* Bruzelius, 1859, *N. buchi* Andres, 1975, *N. recticaudata* Matsukami, Nakano & Tomikawa, 2017, *N. rogeri* Lörz & Schnabel, 2015, and *N. unidentata* Barnard, 1932 (Barnard and Karaman 1991; Lörz and Schnabel 2015; Matsukami et al. 2017). *Nicippe tumida*, the type species of the genus, is a widespread species occurring throughout Arctic-boreal regions (Karaman 1974; Lincoln 1979; Barnard and Karaman 1991). However, recent morphological and molecular research indicates that *N. tumida* is probably a complex of cryptic species (Matsukami et al. 2017).

*Nicippe tumida* was originally described from Drøbak, Norway (Bruzelius 1859). Although the original description was well written and detailed, from a modern taxonomic point of view, it is limited and the figures are restrictive. As several cryptic species may be confused with *N. tumida*, redescription of this species based on topotypic material would aid its differentiation from congeneric taxa.

During an expedition to the Bering Sea, one of us (KK) collected specimens of an evidently undescribed species of *Nicippe* from depths between approximately 500 m. These specimens are described and illustrated here. We also take this opportunity to describe specimens attributed to *N. tumida* from collections of the Swedish Museum of Natural History (SMNH); these specimens were collected from Fredrikshald, Norway, from near the type locality of *N. tumida*.

## Material and methods

### Samples

Specimens of an undescribed species of *Nicippe* were collected from the Bering Sea by R/V *Mirai* (JAMSTEC) in 2017 (MR17-04 Leg2) using a multiple corer or dredge attached on a deeptow camera system. Samples were fixed and preserved in 80% ethanol aboard the ship.

Eight adult females and four juveniles (sex indeterminate) of *N. tumida* from collections of the Swedish Museum of Natural History (SMNH-162979) were borrowed for comparison; one female was dissected (with permission) for detailed description.

### Morphological observation

Appendages were dissected in 80% ethanol and mounted using gum chloral medium on glass slides with the aid of a stereomicroscope (Olympus SZX7). Specimens were examined by light microscopy (Nikon Eclipse Ni) and illustrated using a camera lucida. Body length was measured to the nearest 0.1 mm from the tip of the rostrum to the base of the telson along the dorsal curvature. Type materials have been deposited in the Tsukuba Collection Center of the National Museum of Nature and Science, Tokyo (NSMT).

### PCR and DNA sequencing

Genomic DNA was extracted from pereopod muscle of the holotype and one paratype of the *Nicippe* specimens described herein following procedures detailed in Tomikawa et al. (2014). The primer set for the cytochrome *c* oxidase subunit I (COI) gene (LCO1490 and HCO2198; Folmer et al. 1994) was used for PCR and cycle sequencing reactions. PCR and sequencing followed methods detailed in Tomikawa et al. (2017). The DNA sequence has been deposited with the International Nucleotide Sequence Database Collaboration (INSDC) through the DNA Data Bank of Japan (DDBJ).

## Systematics

### Family Pardaliscidae Boeck, 1871

**Genus *Nicippe* Bruzelius, 1859**

#### Nicippe beringensis sp. nov.

Taxon classificationAnimaliaAmphipodaPardaliscidae

69B24D5E-94EE-5E6E-BE72-3BC04AB42C61

http://zoobank.org/0FAD340E-6303-4B7B-9228-6E38CA8507BD

**Figure 1.**
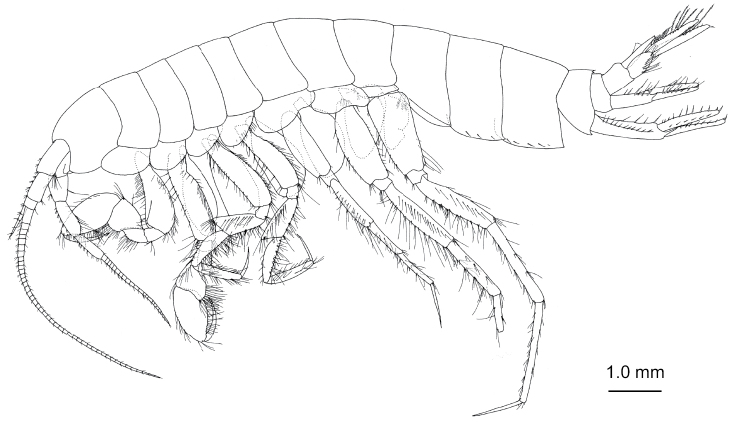
*Nicippe beringensis* sp. nov., holotype female (11.7 mm), NSMT-Cr 27383. Habitus, lateral view.

**Figure 2.**
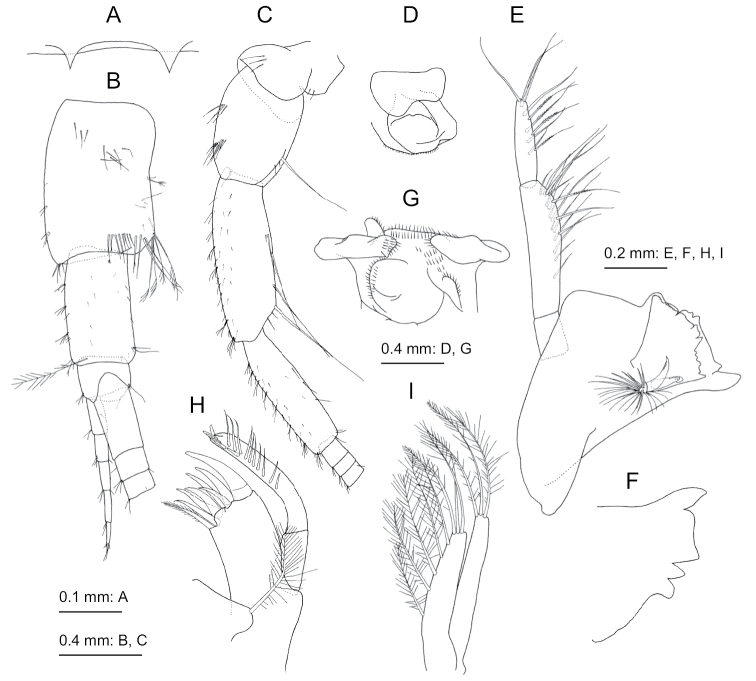
*Nicippe beringensis* sp. nov., holotype female (11.7 mm), NSMT-Cr 27383 **A** dorsal margin of urosomite 1, dorsal view **B** antenna 1, lateral view, some distal articles of primary flagellum omitted **C** antenna 2, lateral view, some distal articles of flagellum omitted **D** upper lip, anterior view **E** left mandible, medial view **F** right mandible, medial view, accessory setal row a proximal tuft of seta omitted **G** lower lip, ventral view **H** maxilla 1, dorsal view **I** maxilla 2, dorsal view.

**Figure 3.**
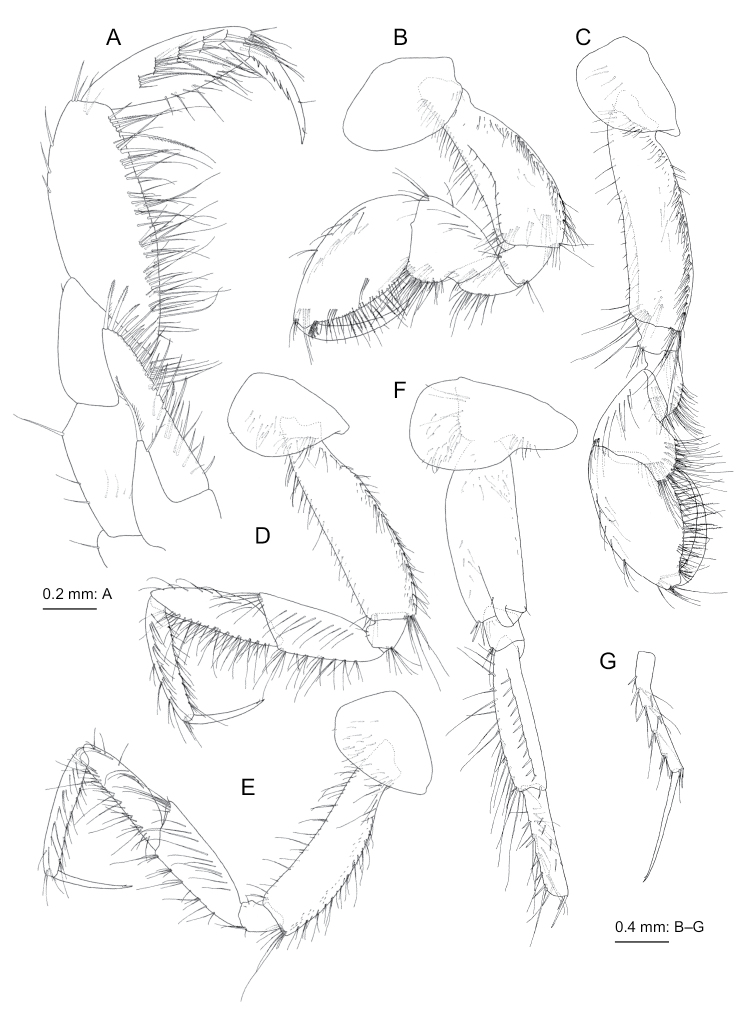
*Nicippe beringensis* sp. nov., holotype female (11.7 mm), NSMT-Cr 27383 **A** maxilliped, dorsal view **B** gnathopod 1, lateral view **C** gnathopod 2, lateral view **D** pereopod 3, lateral view **E** pereopod 4, lateral view **F** coxa-carpus of pereopod 5, lateral view **G** propodus and dactylus of pereopod 5, lateral view, propodus deformed due to preparation.

**Figure 4.**
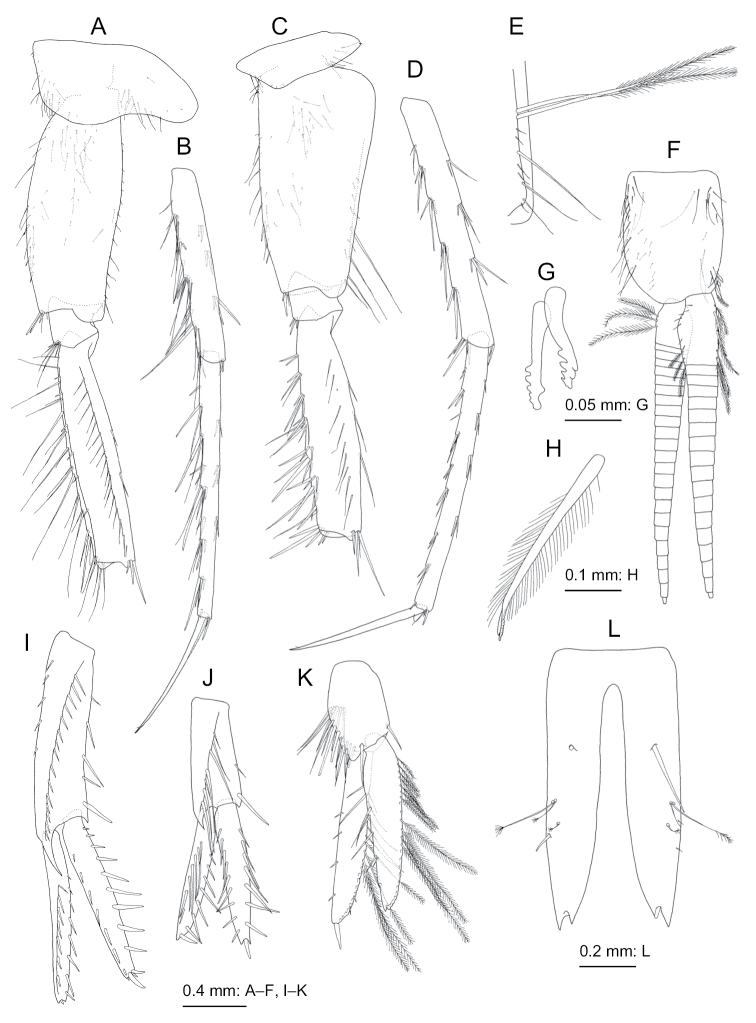
*Nicippe beringensis* sp. nov., holotype female (11.7 mm), NSMT-Cr 27383 **A** coxa-merus of pereopod 6, lateral view **B** carpus-dactylus of pereopod 6, medial view **C** coxa-merus of pereopod 7, lateral view **D** carpus-dactylus of pereopod 7, lateral view **E** posterodistal corner of basis of pereopod 7, medial view **F** pleopod 1, anterior view, some setae on rami omitted **G** retinacula on peduncle of pleopod 1, anterior view **H** bifid plumose seta (clothes-pin seta) on inner basal margin of inner ramus of pleopod 1, anterior view **I** left uropod 1, dorsal view **J** left uropod 2, dorsal view **K** left uropod 3, dorsal view **L** telson, dorsal view.

**Figure 5.**
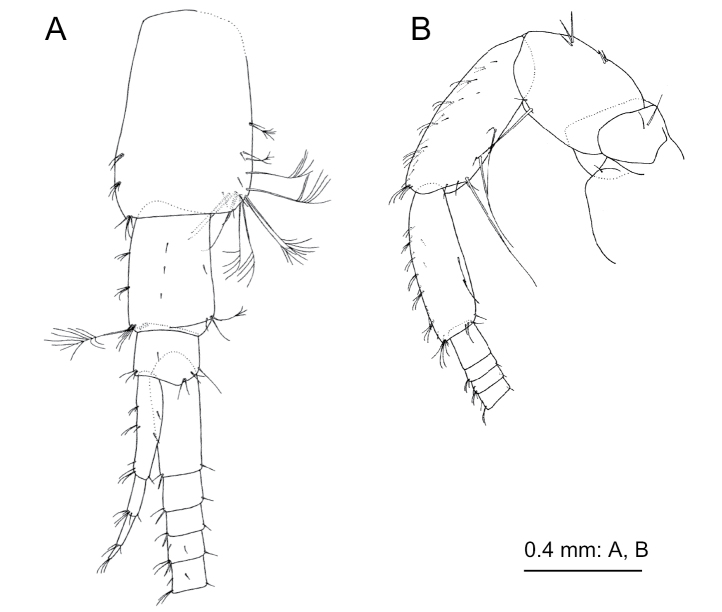
*Nicippe beringensis* sp. nov., paratype male (10.4 mm), NSMT-Cr 27384 **A** antenna 1, medial view, some distal articles of primary flagellum omitted **B** antenna 2, lateral view, some distal articles of flagellum omitted.

**Figure 6.**
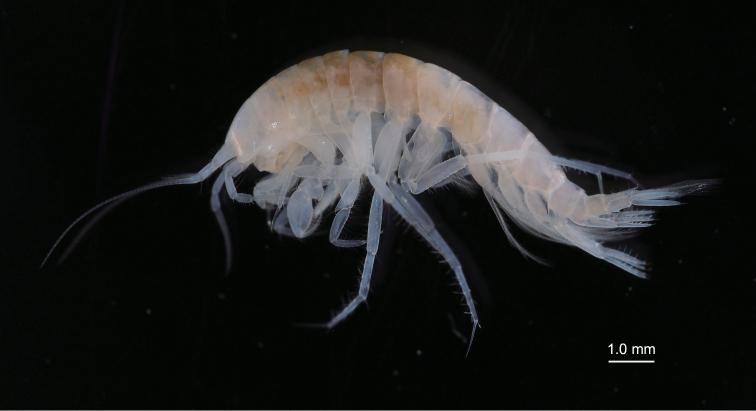
*Nicippe beringensis* sp. nov., paratype male (10.4 mm), NSMT-Cr 27384, in life.

Figures 1 , 2 , 3 , 4 , 5 , 6

##### Material examined.

***Holotype***: ovigerous female (11.7 mm), NSMT-Cr 27383, the Bering Sea (54°28.41'N, 166°01.43'W; Sta. B), 536 m depth, multiple corer, collected by K. Kakui on 6 August 2017. Paratypes: male (10.4 mm), NSMT-Cr 27384, two females (7.8 mm, NSMT-Cr 27385; 6.8 mm, NSMT-Cr 27386), the Bering Sea (54°29.10'N, 165°57.25'W–54°29.08'N, 165°57.23'W; Sta. B), 521–520 m depth, deep tow, collected by K. Kakui on 7 August 2017.

##### Diagnosis.

Urosomite 1 with 2 dorsal spines. Maxilla 1 with asetose palp article 1. Gnathopod 1 coxa with straight distal edge. Basis of gnathopods 1 and 2 and pereopod 3 in female with heavily setose posterior margin. Telson widely cleft with sinuous inner margin, weakly expanding proximally.

##### Description of female

**(holotype, NSMT-Cr 27383). *Head*** (Fig. 1) with rudimentary eye, vertically deep, yellowish, only visible in living specimen (Fig. 6; paratype, NSMT-Cr 27384); rostrum short, pointed; lateral cephalic corner weakly produced. Pereonites 1–7 and pleonites 1–3 dorsally smooth (Fig. 1). Posteroventral corners of epimeral plates 1–3 pointed (Fig. 1); ventral submargins of epimeral plates 2 and 3 with 3 and 5 setae, respectively. Urosomite 1 with 2 pointed spines extending beyond posterior margin of its segment (Fig. 2A); urosomites 2 and 3 dorsally smooth.

***Antenna 1*** (Fig. 2B): length 0.5 times as long as body length; peduncular articles 1–3 in length ratio of 1.0 : 0.8 : 0.3; posterodistal corner of peduncular article 1 with long setae, some of which weakly plumose; accessory flagellum 6-articulate, article 1 short, length 2.5 times of width; primary flagellum 43-articulate, length of article 1 3.0 times as long as article 2.

***Antenna 2*** (Fig. 2C): length 0.7 times as long as antenna 1; peduncular articles 3–5 in length ratio of 1.0 : 1.5 : 1.2; anterior margin of peduncular article 3 with 2 clusters of setae; anterior margins of peduncular articles 4 and 5 with short setae, posterior margin of peduncular article 4 with long setae; flagellum 23-articulate.

***Mouthparts*.** Upper lip (Fig. 2D) with weakly convex ventral margin, bearing fine setae. Mandibles (Fig. 2E, F): slightly asymmetric, incisor margins broad, anteroventral corner with a strong tooth; left lacinia mobilis (Fig. 2E) broad, about 0.8 times as long as incisor, multi-dentate; right incisor (Fig. 2F) with 2 teeth on proximal to anterodorsal corner; right lacinia absent; accessory setal row of left and right mandibles with 2 robust setae, respectively, and a proximal tuft of seta; molar absent; mandibular palp 3-articulate with length ratio of 1.0 : 3.3 : 2.6, article 2 with 5 marginal and 12 submarginal posterolateral setae, article 3 with 7 posterolateral and 3 apical setae. Lower lip (Fig. 2G) with broad outer lobes, inner lobes coalesced. Maxilla 1 (Fig. 2H) with inner and outer plates and palp; inner plate small with apical plumose seta; outer plate subrectangular with 8 apical robust setae, the lateral one strongest and longest; palp 2-articulate, article 1 lacking marginal setae, article 2 expanded distally with robust and slender setae. Maxilla 2 (Fig. 2I) with inner plate bearing row of 5 plumose setae on medial margin and 3 apical plumose setae; outer plate slightly longer than inner plate with 3 apical plumose setae. Maxilliped (Fig. 3A) with inner and outer plates and palp; inner plate not reaching base of palp, with long plumose apical seta and short weakly plumose subapical seta; outer plate narrow, reaching base of article 2 of palp, with setae along apical to medial margin; palp 4-articulate, long, article 2 longest with inner marginal rows of setae, article 3 with clusters of setae on dorsal and ventral faces and inner marginal setae, article 4 slender with serrate inner margin.

***Gnathopod 1*** (Fig. 3B): coxa short, length 1.3 times of width, distal edge straight; basis long, expanded distally, anterior margin flat, posterior margin arched with numerous setae in row; ischium short, subquadrate, 0.7 times as long as merus; posterior margin of merus with setae; carpus with rounded lobe ventrally with long setae, length 1.1 times of width; propodus oval, length 1.8 times as long as carpus, palm weakly convex with long setae; dactylus slender, slightly curved, posterior margin smooth with tooth near the base.

***Gnathopod 2*** (Fig. 3C): coxa rounded ventrally, posterior margin and medial face with setae; compared to that of gnathopod 1, basis longer and more slender; carpus ventrally more strongly produced, length 1.3 times of width; dactylus similar to that of gnathopod 1.

***Pereopod 3*** (Fig. 3D): coxa rounded ventrally, posterior margin and medial face with setae; basis long, posterior margin strongly setose; merus, carpus, propodus, and dactylus in length ratio of 1.0 : 1.0 : 1.0 : 0.6; merus with 9 setae on anterior submargin; carpus with 9 setae on anterior margin and submargin; propodus with 22 setae on anterior margin and submargin; dactylus slender and weakly curved.

***Pereopod 4*** (Fig. 3E): similar to pereopod 3, merus, carpus, propodus, and dactylus in length ratio of 1.0 : 0.9 : 0.9 : 0.6; merus with 10 setae on anterior submargin; carpus with 10 setae on anterior margin and submargin; propodus with 13 setae on anterior submargin.

***Pereopod 5*** (Fig. 3F, G): coxa bilobate with facial setae medially, anterior lobe stronger and longer; basis with posterodistal lobe, anterodistal corner with robust setae; merus, carpus, propodus, and dactylus in length ratio of 1.0 : 0.7 : 0.8: 0.7; merus with 16 slender and 5 robust setae on anterior margin and 12 robust setae on posterior ridge, anterodistal corner with 5 slender and 1 robust setae; posterior ridges of carpus and propodus with 4 and 3 robust setae, respectively; dactylus slender and almost straight, longer than on pereopods 3 and 4.

***Pereopod 6*** (Fig. 4A, B): coxa shallowly bilobate with setae on medial face, anterior lobe slightly stronger; basis posterior margin slightly convex, anterior and posterior margins with setae, anterodistal corner with slender and robust setae, posterodistal corner subquadrate; merus, carpus, propodus, and dactylus in length ratio of 1.0 : 0.8 : 1.1: 0.6; anterior margin of merus with 20 slender and 6 robust setae, posterior margin with 2 setae, posterior ridge with 17 setae; posterior margins of carpus and propodus each with 3 clusters of robust setae; dactylus slender, slightly curved.

***Pereopod 7*** (Fig. 4C–E): coxa subquadrate, anterior margin and ventral submargin with setae; basis distally tapering, posteroproximal part expanded, posterior margin almost straight, anterior and posterior margins with setae, anterodistal corner with robust and slender setae, posterodistal submargin with long plumose setae (Fig. 4E), posterodistal corner rounded; merus, carpus, propodus, and dactylus in length ratio of 1.0 : 1.1 : 1.2 : 0.6; anterior margin of merus with clusters of robust setae, posterior margin with 3 slender setae, posterior ridge with 9 slender setae and trace of seta; anterior and posterior margins of carpus with 5 and 3 clusters of setae, respectively; anterior margin of propodus with 7 clusters of setae and single seta, posterior margin with 3 clusters of setae and single seta; dactylus slender, almost straight.

***Pleopods 1–3*** (Fig. 4F–H) each with paired retinacula (Fig. 4G) on inner distal margin of peduncle, bifid setae (clothes-pin setae) (Fig. 4H) on inner basal margin of inner ramus; inner and outer rami of each pleopod consisting of 18–19 and 23–24 articles, respectively.

***Uropods*.** Uropod 1 (Fig. 4I): peduncle longer than rami, with 5 basofacial setae, distolateral peduncular spine very strong; inner ramus subequal to outer ramus in length, medial and lateral margins with 9 and 7 robust setae, respectively; outer ramus with 7 robust setae on medial and lateral margins, respectively; both rami with stout setae apically. Uropod 2 (Fig. 4J): peduncle slightly shorter than rami, with long stout setae on lateral margin, distolateral peduncular spine short; inner ramus slightly longer than outer ramus, with 7 and 9 robust setae on medial and lateral margins, respectively; outer ramus with 8 and 7 robust setae on medial and lateral margins, respectively; both rami each with stout seta apically. Uropod 3 (Fig. 4K): peduncle strongly setose on lateral face; both rami almost equal in length, medial margins of inner and outer rami with long plumose setae.

***Telson*** (Fig. 4L) length 2.2 times of width, cleft for 89% of length in U-shape with weakly curved margins of incision; apex of each lobe incised, lateral part of apex slightly longer than medial part, with small robust seta.

16 eggs.

##### Description of male

**(paratype, NSMT-Cr 27384). *Antenna 1*** (Fig. 5A): peduncular articles 1–3 in length ratio of 1.0 : 0.6 : 0.3; accessory flagellum 4-articulate, article 1 long, length 4.3 times of width; primary flagellum 38-articulate, length of article 1 3.5 times as long as article 2.

***Antenna 2*** (Fig. 5B): peduncular articles 3–5 in length ratio of 1.0 : 1.3 : 1.1; flagellum 24-articulate.

***Mandible***: palp article 2 with 11 setae; palp article 3 with 5 posterolateral and 3 apical setae.

***Gnathopods 1 and 2***: bases with a few setae on their posterior margins.

##### Etymology.

The specific name is an adjective derived from its type locality, the Bering Sea.

##### Sequences and COI genetic distances.

A single nucleotide sequence was obtained from each specimen of the holotype, NSMT-Cr 27383 (LC511042; 615 bp) and a paratype, NSMT-Cr 27384 (LC511043; 658 bp). Based on the 615 bp aligned sequences, the uncorrected *p*-distances between these two specimens is 0.2%.

##### Remarks.

Though the holotype and paratypes were obtained from slightly different localities, they differ in no appreciable morphological character or state. The pairwise sequence divergence of COI between the holotype and paratype, 0.2%, is considerably less than that of interspecific distances between other amphipod taxa, 3.5–4.0% (Witt et al. 2006; Rock et al. 2007; Hou et al. 2009). Accordingly, we consider these two specimens to be conspecific.

*Nicippe beringensis* sp. nov. is similar to *N. tumida* in having two spines on the dorsal margin of urosomite 1, and in having a telson with a sinuous inner margin that weakly expands proximally. However, our species differs from *N. tumida* in a combination of characters and states: article 1 of the palp on the maxilla 1 is asetose in our new species, whereas it has marginal setae in *N. tumida*; the coxa of gnathopod 1 has a straight distal edge, as opposed to a curved one in *N. tumida*; and the basis of female gnathopods 1, 2 and pereopod 3 has a heavily setose posterior margin (whereas this is sparsely setose in *N. tumida*).

#### Nicippe tumida

Taxon classificationAnimaliaAmphipodaPardaliscidae

Bruzelius, 1859

DF55DB5E-24E0-5574-A58A-F221F1318CB2

**Figure 7.**
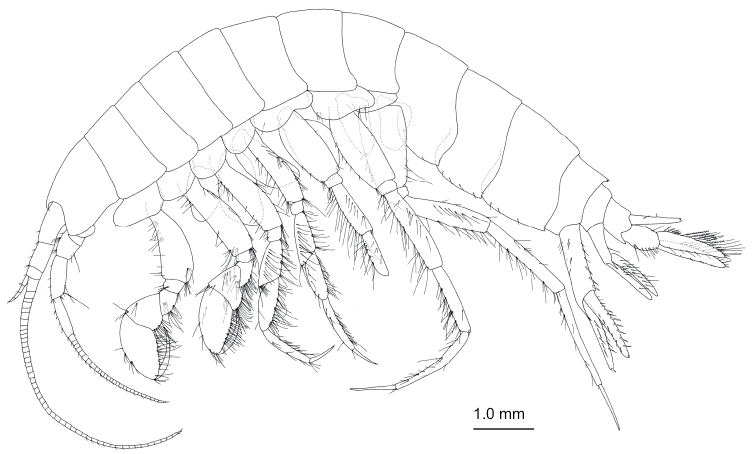
*Nicippe tumida* Bruzelius, 1859, female (12.1 mm), SMNH-162979. Habitus, lateral view.

**Figure 8.**
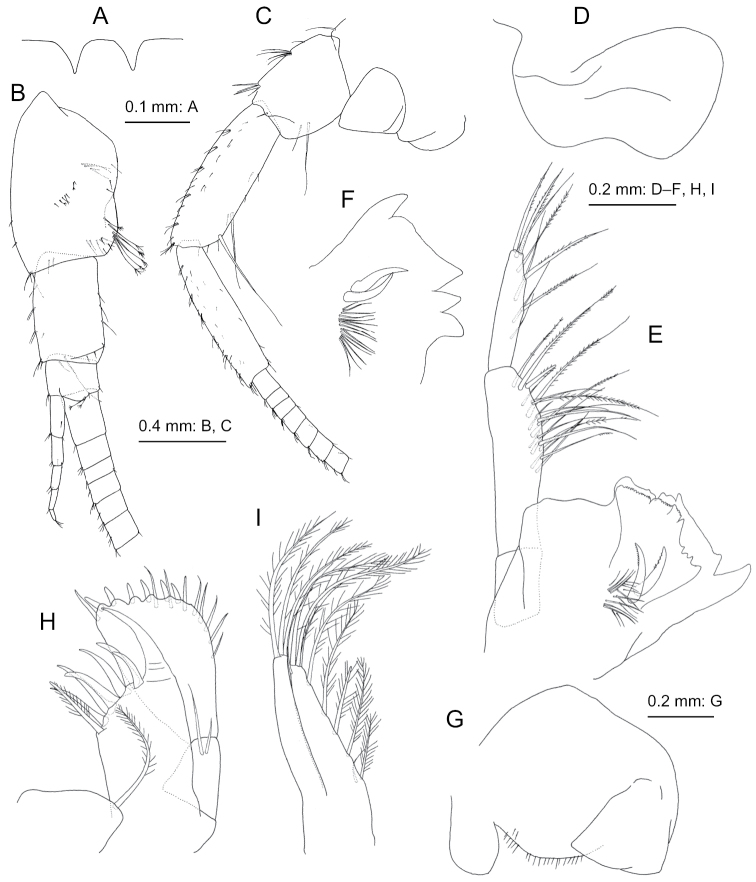
*Nicippe tumida* Bruzelius, 1859, female (12.1 mm), SMNH-162979 **A** dorsal margin of urosomite 1, dorsal view **B** antenna 1, medial view, some distal articles of primary flagellum omitted **C** antenna 2, medial view, some distal articles of flagellum omitted **D** upper lip (labrum broken), anterior view **E** left mandible, medial view **F** right mandible, medial view **G** lower lip, ventral view **H** maxilla 1, dorsal view **I** maxilla 2, dorsal view.

**Figure 9.**
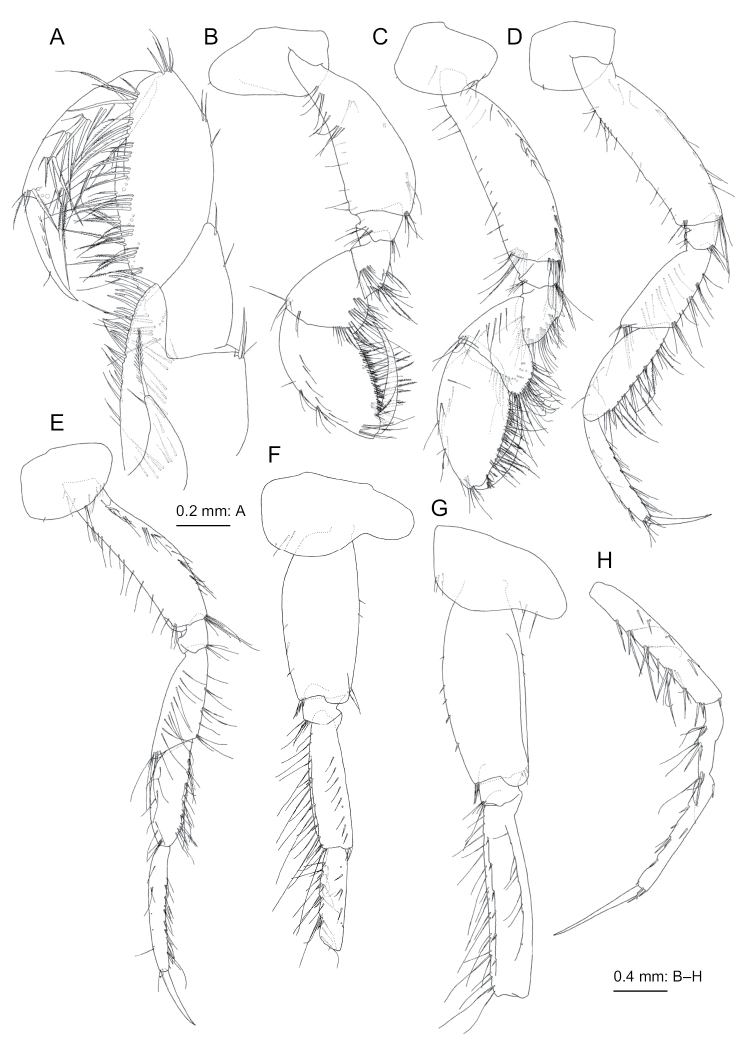
*Nicippe tumida* Bruzelius, 1859, female (12.1 mm), SMNH-162979 **A** maxilliped, dorsal view **B** gnathopod 1, medial view **C** gnathopod 2, lateral view **D** pereopod 3, medial view; **E** pereopod 4, lateral view **F** coxa-carpus of pereopod 5, lateral view, distal part of carpus broken **G** coxa-merus of pereopod 6, lateral view **H** carpus-dactylus of pereopod 6, lateral view, propodus deformed due to preparation.

**Figure 10.**
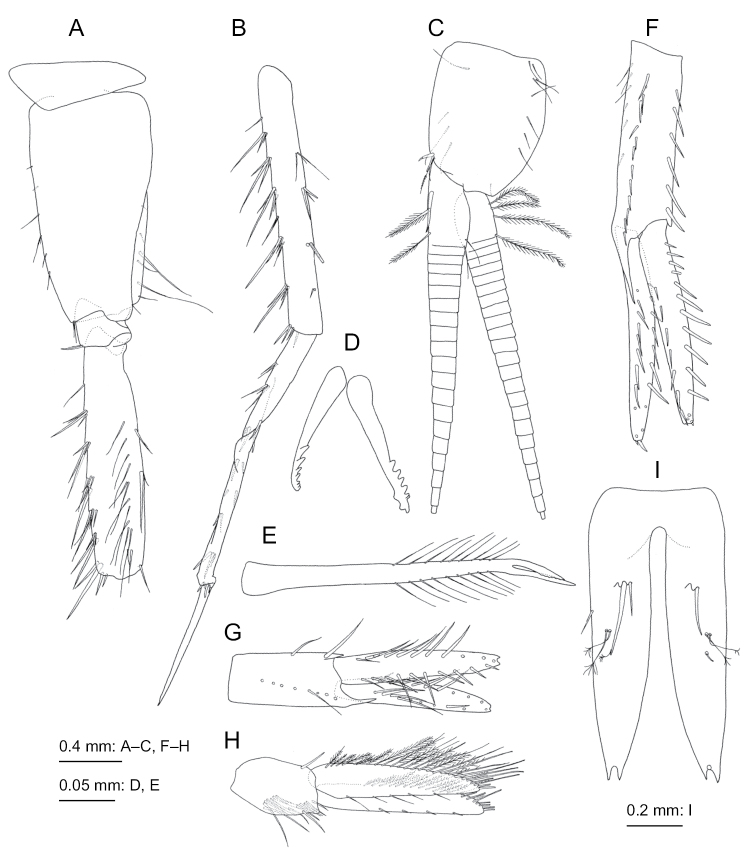
*Nicippe tumida* Bruzelius, 1859, female (12.1 mm), SMNH-162979 **A** coxa-merus of pereopod 7, lateral view **B** carpus-dactylus of pereopod 7, lateral view, propodus deformed due to preparation **C** pleo **E** bifid plumose seta (clothes-pin seta) on inner basal margin of inner ramus of pleopod 1, posterior view **F** left uropod 1, dorsal view **G** left uropod 2, dorsal view **H** left uropod 3, dorsal view **I** telson, dorsal view.

Figures 7 , 8 , 9 , 10

##### Material examined.

Eight females, 11.8–15.0 mm, 4 juveniles (sex indeterminate), 5.8–7.3 mm, SMNH-162979, Fredrikshald, Norway, collected by C. Bovallius in 1869 (the month and date unknown).

##### Description of female

**(12.1 mm, SMNH-162979).** Epimeral plates 1, 2, and 3 (Fig. 7) with ventral submargins bearing 1, 4, and 3 setae, respectively. Urosomite 1 with 2 pointed spines (Fig. 8A)

***Antenna 1*** (Fig. 8B): peduncular articles 1–3 in length ratio of 1.0 : 0.6 : 0.3; accessory flagellum 5-articulate; primary flagellum 48-articulate, length of article 1 1.9 times as long as article 2.

***Antenna 2*** (Fig. 8C): length 0.6 times as long as antenna 1; peduncular articles 3–5 in length ratio of 1.0 : 1.7 : 1.5; flagellum 28-articulate.

***Mouthparts*.** Mandibles (Fig. 8E, F): left lacinia mobilis (Fig. 8E) broad, about 0.7 times as long as incisor; mandibular palp 3-articulate with length ratio of 1.0 : 2.4 : 1.7, article 2 with 20 setae, article 3 with 6 posterolateral and 3 apical setae. Maxilla 1 (Fig. 8H) with palp article 1 bearing 2 marginal setae. Maxilla 2 (Fig. 8I) with inner plate bearing 4 apical plumose setae.

***Gnathopod 1*** (Fig. 9B): coxa broad, length 1.8 times of width, distal edge curved; basis with posterior margin sparsely setose; ischium 0.6 times as long as merus.

***Gnathopod 2*** (Fig. 9C): basis with sparsely setose posterior margin.

***Pereopod 3*** (Fig. 9D): coxa rounded ventrally, ventral margin with seta; merus, carpus, propodus, and dactylus in length ratio of 1.0 : 1.1 : 1.1 : 0.6; carpus with 6 setae on anterior submargin; propodus with 7 setae and traces of 4 setae on anterior margin and submargin.

***Pereopod 4*** (Fig. 9E): merus, carpus, propodus, and dactylus in length ratio of 1.0 : 0.5 : 0.6 : 0.3; merus with 11 setae on anterior submargin; carpus with 7 setae on anterior margin and submargin; propodus with 4 setae and traces of 3 setae on anterior margin and submargin; dactylus slender and weakly curved.

***Pereopod 5*** (Fig. 9F): basis with 3 and 2 setae on anterior and posterior margins, respectively, posterodistal corner of basis subquadrate; merus with 16 long and 3 short setae on anterior margin and submargin, 8 setae and trace of seta on posterior ridge, anterodistal corner with 3 setae.

***Pereopod 6*** (Fig. 9G, H): coxa shallowly bilobate with setae on ventral margin and submargin; basis posterior margin almost straight, anterior and posterior margins with 6 and 2 setae, respectively, posterodistal corner of basis rounded; merus, carpus, propodus, and dactylus in length ratio of 1.0 : 0.8 : 1.0: 0.5; merus with 21 setae on anterior margin, 7 short setae on anterior submargin, 6 setae and traces of 4 setae on posterior ridge; posterior margins of carpus and propodus each with 5 (+ traces of 2 more setae) and 2 robust setae; dactylus slender, almost straight.

***Pereopod 7*** (Fig. 10A, B): merus, carpus, and propodus in length ratio of 1.0 : 1.1 : 1.1: 0.5; merus posterior ridge with 13 setae.

***Pleopods 1–3*** (Fig. 10C–E): inner and outer rami of each pleopod consisting of 19–20 and 22–24 articles, respectively.

***Uropods*.** Uropod 1 (Fig. 10F): peduncle with 6 basofacial setae; inner ramus slightly shorter than outer ramus, medial and lateral margins with 11 and 7 robust setae, respectively; outer ramus with 7 robust setae and traces of setae on medial and lateral margins, respectively. Uropod 2 (Fig. 10G): inner ramus with 8 (+ traces of 2 more setae) and 10 robust setae on medial and lateral margins, respectively; outer ramus with 7 and 5 robust setae on medial and lateral margins, respectively and traces of setae.

***Telson*** (Fig. 10I) length 2.1 times of width, cleft U-shaped, with weakly curved margins of incision.

##### Remarks.

Though the present specimens agree with the original description of *N. tumida* (Bruzelius 1859), there are some differences between them. Pereopod 7 bears long plumose setae on the posterior margin of the basis in the original description, whereas the present specimens lack these setae. We confirmed that these plumose setae are fragile and easily broken off during handling for the observation of *N. beringensis*. Thus, these setae may have been broken off in the present specimens. The telson of the present material takes on the almost parallel shape at either inner margin. Judging from the illustration of the original description, the telson has a wide cleft distally and its inner margins are not parallel. It is a highly likely that the telson was erroneously drawn because Bruzelius’ illustrations were simplified and there are some unreasonable aspects, e.g. the shape of coxae of pereopods 5–7 and epimeral plates. To confirm these dubious features as well as the telson shape, examination of the type material is needed. In this study, we could not access the type materials of *N. tumida*. Unfortunately, it is likely that the type specimens are lost.

### Key to species of *Nicippe*

**Table:** 

1	Dorsal margin of urosomite 1 smooth	***N. buchi***
–	Dorsal margin of urosomite 1 dentate	**2**
2	Dorsal margin of urosomite 1 with 1 spine	**3**
–	Dorsal margin of urosomite 1 with 2 spines	**4**
3	Pereonites 1 and 7 the longest, pereonites 2–6 shorter, subequal; uropod **3** peduncle with unarmed distodorsal lobe	***N. rogeri***
–	Pereonites 1–4 subequal, shorter than pereonites 5–7 with segment 5 the longest, uropod 3 peduncle with 3 pointed distodorsal processes	***N. unidentata***
4	Telson narrowly cleft with straight inner margin, tapering proximally	***N. recticaudata***
–	Telson widely cleft with sinuous inner margin, weakly expanding proximally	**5**
5	Maxilla 1 with asetose palp article 1; gnathopod 1 coxa with straight distal edge; basis of female gnathopods 1 and 2, and pereopod 3, with heavily setose posterior margin	***N. beringensis***
–	Maxilla 1 with palp article 1 bearing setae; gnathopod 1 coxa with curved distal edge; basis of female gnathopods 1 and 2, and pereopod 3, with sparsely setose posterior margin	***N. tumida***

## Supplementary Material

XML Treatment for Nicippe beringensis

XML Treatment for Nicippe tumida
